# Supplementary material for: Are carbon emissions trading and green financial instruments synergistic? -Comprehensive quantitative research based on content analysis
Source: PLoS One. 2024 Mar 7;19(3):e0298601. doi: 10.1371/journal.pone.0298601 (PMC10919601; doi:10.1371/journal.pone.0298601)
Supplement: S1 Data — (ZIP) [file pone.0298601.s001.zip › Data/地级市Z_F工作报告.zip!/╣≤╓▌/┴∙┼╠╦«╩╨/└┤╫╘╣1⁄2╓┌║┼í╛┬φ┐╦╩2╛▌═°í┐_2005.docx]

各位代表：

　　现在，我代表市人民政府，向大会作工作报告，请予审议。并请各位政协委员和列席的同志提出宝贵意见。

2004年政府工作回顾

　　过去一年，我们坚持以邓小平理论和“三个代表”重要思想为指导，认真贯彻落实党的十六大和十六届三中、四中全会精神，全面落实科学发展观和国家宏观调控政策，切实把加快发展作为第一要务，求真务实，能快则快，扎实工作，开拓创新，全市经济社会全面快速发展，市五届人大四次会议确定的各项目标任务圆满完成。

　　（一）经济增长速度明显加快，运行质量和效益进一步提高。生产总值完成151.1亿元，增长18.2%，一产、二产、三产分别增长5.6%、24.2%和12.6%。全社会固定资产投资完成85.69亿元，增长60.5%。规模以上工业企业实现利润总额4.49亿元，增长13%；社会消费品零售总额完成29.4亿元，增长20.1%。财政总收入按老口径计算完成20.52亿元，增长34.47%，按新口径计算完成19.2亿元，增长33.85%，其中地方财政收入完成9.7亿元，增长32.62%。金融机构存贷双增，金融机构支持地方经济社会发展的力度进一步加大，各项存款、贷款余额分别为168.59亿元和112.37亿元，分别比年初增加40.36亿元和22.01亿元，净投放现金21.55亿元，较上年增加5.68亿元。生产总值、财政总收入、固定资产投资等三项主要指标的增幅连续两年在全省各市、州、地中名列第一。

　　（二）“三农”工作力度加大，农业和农村经济进一步发展。认真贯彻2004年中央和省、市三个“一号文件”精神，全面落实农业“两免一补”等各项支农政策。主要农产品全面增产，粮食产量再创历史新高，达75.82万吨，增长4.8%。畜牧业增加值扣除物价因素实际增长16.1%，畜牧业产值占农业总产值的比重升至33.8%。烤烟生产实现恢复性增长，收购量达9590吨，增长86.1%，担均价提高了89.12元。乡镇企业增加值完成43.62亿元，增长26%。扶贫开发力度进一步加大，各级投入的财政扶贫资金共达5203.72万元，新增发放农村小额扶贫贷款5063万元，又有8万人越过温饱线；帮助6个一类重点贫困乡偿还了长期拖欠的政府性债务。农民人均纯收入1672.12元，增收170.14元，扣除物价因素实际增长6.8%。

　　（三）西部大开发重点项目扎实推进，加快发展的基础进一步夯实。全面贯彻国家宏观调控政策，按照有保有压的原则，一方面开展固定资产投资项目清理和土地市场秩序治理整顿，遏制了低水平重复建设和盲目投资；另一方面，紧紧抓住重点项目建设这个关键，千方百计扩大投资规模。全年市、县两级投入项目前期工作经费3557万元，增长46.4%。继续实施政银合作，共到位资金3.65亿元。盘南电厂、发耳电厂、野马寨电厂等重点项目建设进展顺利，分别累计完成投资27.12亿元、10.8亿元和11.96亿元。响水、发耳、玉舍等煤矿建设顺利，玉舍煤矿首采工作面进入试产。镇胜高速公路境内段全线动工，组建贵州红威公路有限责任公司并全面启动了红威高等级公路建设；改造公路245公里，建成通村公路1000公里。开通了水柏铁路客运。解决了11.09万人、4.51万头牲畜的饮水困难，还建成了一批“烟水工程”和与坡改梯配套的小水池。建成沼气池6500口。完成17.5万亩人工造林和3万亩封山育林任务，石漠化治理试点正式启动，全市森林覆盖率升至26%。“十件实事”基本完成。

　　（四）经济结构不断优化，主导产业对经济增长的贡献进一步加大。支持企业加大技改投入，实施了16个重点技改项目，全年完成技改投入21亿元，增长11.2%。水城矿业（集团）公司老矿井千万吨技改启动，水城钢铁（集团）公司3号高炉建成投产，水城水泥股份有限公司日产2000吨新型干法生产线技改建成试产。规模以上工业增加值完成54.61亿元，增长20.5%，其中煤炭、钢铁、电力、建材四大行业对工业增长的贡献率达84.9%。切实把煤化工作为煤炭产业的发展方向，开展了《六盘水市煤化工基地发展规划》编制工作，120万吨西洋焦化、90万吨旗盛焦化等一批大型煤焦化项目开工建设。正式启动了“地企共建”。非公有制经济发展加快，非公有制经济在生产总值中的比重提高了2个百分点，达27.5%。旅游总收入完成1.53亿元，增长135%。

　　（五）城市建设步伐明显加快，城市整体形象进一步提升。理顺了市中心城区城市管理权及“三角区”行政管理关系，将钟山经济开发区委托钟山区代管。完成了钟山大道改扩建，启动了凉都大道改造工程。新区建设加快，旧城改造稳步推进，城区面积不断扩大，城镇化水平进一步提高。深入开展文明卫生城市创建活动，城市面貌进一步改观。着力打造“凉都”城市品牌，相继成功举办消夏文化节、金秋乌蒙系列文化活动，承办了全国汽车拉力锦标赛六盘水站比赛、全国甲级围棋联赛六盘水赛区比赛等大型体育赛事，充分展示了我市“三个文明”建设的成果，提高了我市知名度和在区域经济中的影响力。

　　（六）经济体制改革稳步推进，对外开放进一步扩大。国有企业改革取得新进展，水城水泥股份有限公司与香港瑞安集团公司达成合资协议，六盘水华鼎印务公司股份制改造和六盘水卷烟厂关闭破产顺利完成。资产重组后的水红铁路有限责任公司挂牌成立。组建了政府性投资公司，主要受市人民政府委托代建政府性投资项目。财政改革取得积极成效，国库集中支付改革继续推进；市级部门预算改革实施面已达85%；政府采购步入法制化管理轨道。按时完成了政府机构改革和国土资源管理体制改革，启动了水利工程管理体制改革。认真贯彻实施《中华人民共和国行政许可法》，依法取消了一批行政审批事项。积极参与“泛珠三角”等区域经贸活动，不断加强与周边地区的友好往来，招商引资成效显著，全年共到位资金25.76亿元，增长54.1%。

　　（七）各项社会事业继续向前发展，经济社会发展的协调性进一步增强。继续把2004年作为“重教兴教年”，全年对教育的投入达5.05亿元，增长33.5%。盘县“两基”通过省级评估验收，钟山区“两基”复查和“普实”工作经省级验收合格，以乡为单位的“普九”人口覆盖率达93.8%，以县为单位的“普九”人口覆盖率达53.6%。人口与计划生育工作取得新成绩，人口出生率为15.7‰，下降了1.63个千分点；人口自然增长率为9.2‰，下降了1.27个千分点；符合政策生育率达93.33%，上升了11.94个百分点。计划生育“创优”工作全面启动，率先在全省启动育龄妇女妇科病普查普治，全市89.13%的村（居）实现“村为主”，“村为主”工作通过省级验收，六枝特区获国家级计划生育优质服务先进县称号。在全省评比中，市级在党政线、计生线考核中均获一等奖，盘县获突出进步奖。卫生事业加快发展，市、县疾控中心启动建设，着手建立了农村医疗救助制度，6个试点乡新型农村合作医疗试点全面启动，9.8万人受益。双拥模范城创建活动深入开展，民族团结进步事业成效显著，宗教政策得到较好落实，广播、电视、环保、审计、气象、档案、地方志、对台、外事侨务等工作取得新成绩，妇女、儿童、老龄、残疾人和红十字事业继续发展，精神文明建设和民主法制建设不断推进，政府自身建设进一步加强。

　　（八）社会建设和管理得到加强，构建社会主义和谐社会进一步推进。将扩大就业和控制失业纳入市、县两级政府宏观调控的主要目标，提前4个月全面超额完成省下达的就业和再就业任务。新增城镇就业20689人，其中促进下岗失业人员再就业7262人、“4050”人员再就业812人，城镇登记失业率为4.3%。“两个确保”实现100%，“三条保障线”衔接较好。养老保险、失业保险和医疗保险扩面征缴任务全面完成，工伤保险启动实施。提高了城市低保标准，扩大了低保覆盖面，基本实现了“应保尽保”。城镇居民人均可支配收入7176.96元，扣除物价因素实际增长9.9%。全面清理建筑领域拖欠工程款和农民工工资，共为37971名农民工追回工资3499.88万元。农民负担进一步减轻，全市农民负担总减负率为57.47%，人均减负24.46元。大力实行义务教育阶段“一费制”，减轻学生负担402.63万元。积极推行药品集中招标采购和医药分开核算制度，让利于患者578万元。采取减免民用煤税费、成本价供应、运输补贴和民政部门对特困农户实施生活用煤救济等措施，努力解决农村生产生活用煤问题。认真研究解决失地农民长远生计问题，在盘县响水“一厂一矿”搬迁农户中启动试点工作。进一步加大安全生产工作力度，安全生产事故起数和死亡人数在省下达的指标内实现“双降”目标，为全省实现“双降”作出了贡献。救灾救济工作和社会福利事业向前推进，自然灾害救援能力日益增强。全市社会治安综合治理继续保持省的模范称号，禁毒、打黑除恶、反邪教等工作深入开展，刑事案件、治安案件发案率分别下降14.1和9.6个百分点。在全省率先进行市中心城区警务体制改革。信访工作力度加大，信访总量下降5%。

　　2004年经济社会的持续快速发展，为全面完成和超额完成“十五”计划奠定了坚实基础。生产总值、财政总收入、人口自然增长率等经济社会发展的重要指标提前一年实现“十五”计划，西部大开发“五年打好基础”的战略目标基本实现，原预计到2010年实现人均生产总值比2000年翻一番的进程可提前三年完成。

　　各位代表：2004年全市经济社会发展取得的成绩，是省委、省政府和市委正确领导的结果，是各级人大、政协支持和帮助的结果，是全市各族干部群众团结拼搏、共同努力的结果。在此，我代表市人民政府，向全市工人、农民、知识分子、干部和离退休老同志，向各民主党派、工商联、人民团体、无党派人士，向驻市人民解放军、武警官兵、公安干警，向所有关心支持六盘水发展的境内外朋友，表示衷心的感谢并致以崇高的敬意！

　　总结过去一年的工作，我们清醒地看到我市发展中还存在着不少困难和问题，主要是：农业生产基础薄弱，农村贫困面大，城乡二元结构突出；经济增长方式粗放，制约经济社会健康发展的体制性机制性问题仍很突出，经济结构调整任重而道远；煤电运等生产要素供求仍很紧张，经济运行协调难度较大；国有企业改革举步维艰，非公有制经济发展不快；财政收支平衡难度仍然较大；安全生产基础薄弱，形势不容乐观；教育、科技、文化、卫生等社会事业发展相对滞后；影响社会稳定的因素还未消除，维稳工作任务艰巨。同时，我们也清醒地看到政府工作还存在着许多不足，主要表现在：政府改革和职能转变滞后，还不能适应经济社会发展的需要；一些公务人员发挥主观能动性不够，工作不实，效率不高，形式主义、官僚主义的现象还较为严重；关心困难群众生产的工作存在差距，一些关系群众切身利益的问题还没有得到根本解决；腐败案件时有发生，党风廉政建设需要加强。对于存在的问题，我们将高度重视，并采取切实有力的措施逐步加以解决。

2005年政府工作的主要任务

　　今年政府工作总的要求是：以邓小平理论和“三个代表”重要思想为指导，全面贯彻落实党的十六大和十六届三中、四中全会以及全国和省“两会”精神，更加坚定不移地贯彻发展是硬道理，更加坚定不移地贯彻发展这个执政兴国的第一要务，更加坚定不移地树立和落实科学发展观，进一步抓住机遇、用好机遇，继续推进我市社会主义物质文明、政治文明、精神文明建设与和谐社会建设的全面发展，认真贯彻宏观调控政策，紧紧围绕西部大开发和扶贫开发的战略重点，发扬后来居上、勇攀高峰、能快则快、争创一流的精神，着力推进改革开放，不断完善体制机制，加快经济结构调整，切实转变经济增长方式，促进经济社会全面协调可持续发展，确保实现今年经济社会发展的预期目标，全面完成和超额完成“十五”计划的各项任务，努力实现六盘水市经济社会发展的历史性跨越。

　　今年全市经济社会发展的主要预期目标为：生产总值增长13%以上；全社会固定资产投资完成110亿元；财政总收入增长15%以上，其中地方财政收入增长10%以上；居民消费价格总水平控制在104%左右；城镇居民人均可支配收入增长8%；农民人均纯收入增长100元以上；城镇登记失业率控制在4.3%以内；人口自然增长率控制在10‰以内。以上预期目标是预测性、指导性的，同时也是底线，我们将根据发展情况适时进行调整，努力把工作做得更好。

　　围绕实现以上目标，今年政府工作重点抓好以下六个方面：

　　一、加大“三农”工作力度，努力提高农业综合生产能力

　　解决好“三农”问题是一项长期的、艰巨的历史任务，必须坚持把“三农”工作作为重中之重，始终不懈地抓好。把握“两个趋向”，坚持站在经济社会发展全局的高度，统筹城乡经济社会发展，努力做到城市支持农村、工业反哺农业，努力实现城乡良性互动、工农共同发展。

　　认真落实各项支农政策和措施，继续加大对农业的支持力度。坚持“多予、少取、放活”的方针，不断加大支农投入，切实落实农业税全免等支农政策。今年市级财政农业专项资金安排3530万元，其中扶贫专项资金1000万元。各县、特区、区也要相应加大对农业的投入。继续实行对种粮农民的直接补贴，扩大良种补贴范围和品种，加大对购置农机具补贴和农村劳动力职业技能培训等方面的投入。要按照突出重点、相对集中、统筹使用的原则，捆绑使用好各项支农和扶贫资金。积极争取金融机构加大对农村经济社会发展的支持力度，继续组织好小额扶贫贷款的发放工作，努力扩大发放规模。完善涉农价格和收费公示制度，切实减轻农民负担。认真研究并逐步建立落实支农政策和措施的长效机制，使之制度化、规范化。

　　努力提高粮食综合生产能力，下大力稳定粮食生产。加强基本农田保护，确保粮食播种面积稳定在307万亩以上。大力推广普及农业适用技术，着力提高良种良法普及率，努力提高粮食单产，实现全年粮食增产2%的目标。实施“优质粮食产业工程”，强化农业生产技术集成；大力实施“农业科技入户工程”，做到科技到户、良种良法到田、关键技术到人。力争完成55万亩脱毒马铃薯种植。加快马铃薯种薯繁育体系建设。搞好高海拔地区农作物新品种的引种、选育及试验示范。按照强化公益性职能、放活经营性服务的要求，深化乡镇农业科技推广体系改革，认真落实农业科技人员“两个80%”。充分挖掘耕地潜力，培肥地力，累计建成90万亩高产稳产农田。加强防灾减灾工作。切实做好化肥等农用物资的有效供给，加强农资价格和质量监管，有效控制农业生产成本。

　　加快农业结构调整，多渠道开辟农民增收途径。全面实施《六盘水市农业优势特色产业开发规划》，大力发展草食畜牧、茶叶、蔬（野）菜、马铃薯、经济林果、中药材六大特色产业。加快制定农业标准体系。积极实施“无公害食品行动计划”，提高农产品质量。继续抓好烤烟生产，全面完成18万担烟叶生产计划。切实抓好10万亩马铃薯商品基地建设。充分发挥畜牧业对农民增收的主渠道作用，继续抓好盘县优质肉牛基地、水城县奶源基地；扩大人工种草面积，完成人工种草3万亩以上；加强商品肉牛规模育肥工作，全年力争肉类产量增长10%以上，畜牧业产值占农业总产值的比重达到35%左右。加快农产品市场体系建设，争取在牲畜专业市场建设上有实质性的进展。大力发展农产品加工业，加大对龙头企业的扶持力度，积极支持农村专业协会和经济合作组织加快发展，提高农民进入市场的组织化程度，推进农业产业化进程。

　　按照扶贫开发“三个基本”的要求，扎实推进新阶段扶贫开发。加大开发式扶贫工作力度，继续巩固6个一类重点贫困乡的扶贫开发成果，启动实施100个一类重点村扶贫开发整村推进工作。推广参与式扶贫，坚持和完善挂钩扶贫制度和特困群众定期救助制度，努力在全社会形成扶贫合力。落实土地等生产要素，加强配套设施建设，推进易地扶贫搬迁。通过一年的努力，力争基本解决一类贫困人口的温饱问题，完成533户二类贫困人口的搬迁，将三类贫困人口全部纳入民政救济。

　　加强农业基础设施建设，改善农村生产生活条件。以方便群众生产生活为出发点，加大农村基础设施建设力度，抓紧乡村道路、农村小水电、农网改造、广播电视、农经网进村等基础设施建设；在省的支持下，实施并基本完成“村村通电话”工程。加强农田水利建设，着力解决工程性缺水问题。组织实施好基本农田、小型微型水利、农村沼气、土地整治等工程，进一步实施好农业综合开发项目，全面完成4.6万亩坡改梯、1658口小水池、8000口农村沼气池建设任务，解决10万人的饮水困难。继续实施民政安居工程，启动茅草房改造。引导和鼓励农民投工投劳参加直接受益的小型基础设施建设。

　　加大农村劳动力培训力度，提高农村劳动者素质。整合培训资源，大力实施以农业实用技术培训为主的绿色证书培训、以非农技能培训为主的“阳光工程”等“农村能人工程”，培养一批有一定种植养殖水平的农村致富能人和有一定技能的农村外出务工能手。加快建立农村劳动力就业培训和转移统筹协调机制，逐步形成政府主导、统一协调、各方参与的农村劳动力转移培训体系。积极引导农民自主外出务工，并努力增加有组织的农民外出务工。大力推广订单培训，实行定向培训、定点输出，实行培训经费与培训转移就业挂钩。高度重视维护农民工合法权益，逐步取消对农民进城的限制性规定。支持和鼓励工会、共青团、妇联及其他社会组织帮助转移农村劳动力。

　　二、加大固定资产投资力度，拉动经济较快发展

　　切实抓好项目建设，保持固定资产投资合理较快增长。紧紧围绕西部大开发的战略重点，进一步加快以煤电、交通、水利为重点的项目建设，确保全年固定资产投资达到110亿元。煤电建设方面，启动黑塘、土地丫口、松河及格目底矿区中区和东区等矿区开发，加快盘南、发耳等电厂和响水、发耳等煤矿建设进度，建成野马寨电厂和玉舍煤矿，争取六枝电厂、善泥坡电站进场，开工建设松河300万吨焦化项目。交通建设方面，切实做好镇胜高速公路建设、两水公路改造协调服务工作，加快红威公路红果至响水段及响水到大山支线、俄发公路发耳至发耳火车站段、马场经米箩至玉舍公路建设，争取开工新建双水至岩脚公路双水至老鹰山段及化乐至岩脚段、水盘东线野钟至花嘎及羊场至英武段、羊柏线淤泥至柏果段，完成赵官至乐民、汪家寨至木果等一批油路改造工程；建成水普公路和六纳公路及其延伸段，完成水盘东线米箩至野钟段公路改造，建成1387公里通村公路。同时，要高度重视并认真研究解决县乡公路的管护问题。水利建设方面，积极配合省抓好黔中水利枢纽一期工程建设前的准备工作，继续实施病险水库除险加固和水土保持综合治理工程。在项目建设中，要切实加强管理，严格落实“六制”和“三控制”。

　　切实做好项目前期工作，抓好项目库建设。今年市级财政预安排2200万元资金作为项目前期工作经费，较去年增长37.5%。要抓紧做好六沾铁路复线和客站换边、新建货场等项目的前期工作，并积极争取国家尽快开工建设；全力推进毕水兴高速公路前期工作，争取纳入全省“十一五”交通规划；继续做好水资源综合规划，加快推进白河沟水库、岱翁水库、发耳片区万亩农田灌溉工程的前期工作；争取省的支持，积极开展双桥水库、玉舍供水工程第二条输水管道的前期工作；继续开展六盘水支线机场的前期工作；做好以县为单位的石漠化综合治理前期工作。与此同时，要围绕国家国债和财政性建设资金投入的重点，在农村基础设施和重大水利项目建设，基础教育、公共卫生体系、公检法司设施、生态建设和环境保护等领域，抓好项目库建设，争取国家和省加大对我市的投入。

　　加快投资体制改革步伐，尽快改变投资渠道单一的现状。积极引导社会资金投向煤炭、电力、交通、城市基础设施、教育、旅游等领域；利用TOT、BOT、BT等融资方式，大力推进玉龙水厂、污水处理厂、垃圾填埋场的经营权转让；尽快组建路桥投资开发公司。继续开展政银合作、政企合作，积极争取金融部门和融资性投资企业对我市政府性项目进行融资。全面清理土地、矿产、林地和水资源的出让收入及各类专项资金、基金，逐步实行统筹使用。进一步加大招商引资力度，继续开展活动招商、网上招商、会展招商、以商招商等，扩大引资规模，吸引外来资金参与我市经济建设。全面开展存量土地清查特别是可建设用地存量的清查和保护工作，启动市、县两级国有土地储备。

　　加强对各类专项资金特别是重点项目建设资金的管理，提高资金使用效益。合理使用预算安排的专项资金，加快项目安排和资金拨付进度，上半年使用进度要完成一半以上，三季度使用进度要达90%以上。加强重点项目资金审计。抓紧建立项目建设地方配套资金到位激励约束机制，加大项目资金监管和追踪问效，实现对投资项目预算的动态管理、投资进度监测及竣工投产后的效益考核与事后评估，有效遏制建设项目中截留、挪用资金以及概算超估算、预算超概算、决算超预算的现象。

　　三、加快经济结构调整，切实提高经济增长的质量和效益

　　坚持做大做强煤炭、钢铁、电力、建材等支柱产业。在继续抓好“西电东送”及配套项目建设的同时，大力支持水城钢铁（集团）公司、水城矿业（集团）公司、盘江煤电（集团）公司、六枝工矿（集团）公司、水城水泥股份有限公司、双牌铝业公司等企业进行技改扩能，加快创建“百亿企业”和“名优企业”步伐。规划新建一批30万吨以上地方煤矿，技改扩能一批“小改大”的年产30万吨以上的地方煤矿。坚持走新型煤炭工业可持续发展之路，瞄准把煤化工培育成我市又一支柱产业的目标，尽快完成《六盘水市煤化工基地发展规划》，启动盘东煤焦电一体化项目、盘南煤制油项目、煤化工深加工项目和煤层气开发项目的前期工作。

　　优化工业产业结构，进一步加强经济运行调节。发挥能源优势，做好高载能产业规划，争取新上一批符合国家产业政策，上规模、上档次、集约化程度高的高载能项目。按照“产量不减，市场不丢，上大压小，渐次调整”的思路，积极引导高载能企业提高产业集中度和技术装备水平。对市场供不应求、技术含量高、执行环保措施好、节能有成效的高载能企业和产品，给予大力支持。坚持“扬大、弃小、汰劣”的方针，按照“税费从高、处罚从重”的原则，采取政策、法律、行政、经济等措施，逐步压缩改良焦、小机焦的生存空间，引导其自行退出市场，确保在2005年淘汰改良焦，在2007年淘汰小机焦。根据国家产业政策和我市的实际，不再审批炭化室高度在4.3米以下、总生产能力在60万吨以下，不具备焦炉煤气净化利用与煤化工产品回收工艺、无配套装煤除尘和推焦除尘装置等要求的煤焦化项目。积极发展轻工业，重点发展马铃薯等农产品加工业。继续加强经济运行调度，着力解决煤电运紧张的问题。按照“保电煤、保民用煤、保就地转化用煤”的要求，加强对煤炭生产经营的调度。

　　大力发展各类服务业，进一步扩大消费。把旅游业作为服务业的重点来抓，切实加大气候资源、地质遗迹、地貌景观、民族风情等的开发力度。做好六盘水乌蒙山国家地质公园的申报工作；加快玉舍森林旅游开发；启动阿勒河、天生桥景区建设，理顺阿勒河景区开发管理体制；完成??江风景区毛口景区规划，加快以??湖为中心的毛口景区建设。充分发挥交通区位优势，支持钟山区、水城县加快建设市中心城区大型专业物流市场，尽快构建我市物流体系。抓紧完善全市商业网点规划，以商业连锁业、社区服务业为重点，着力发展城市服务业。进一步规范房地产市场，落实好经济适用住房优惠政策。积极发展交通运输业特别是公路运输业，大力支持水城汽车运输公司和市公交公司的改革与发展，支持一些有实力的非公有制经济性质的城市客运、长途客运、煤焦货运、零担货运走公司化、规模化、规范化发展道路，加快提升改造交通运输业。加强农村水电路、邮政、通信等基础设施和农村市场体系建设，促进农村消费。

　　推进城镇化进程，加快培育城市经济。继续按照“绿化、美化、文化和现代化”的要求，努力把我市建成贵州“西部明珠”。强化规划的龙头地位，进一步加强市中心城区的规划、建设、管理。在抓好城市新区和旧城改造详细规划的同时，抓紧启动城市总体规划第三轮修编工作，统筹做好老鹰山、汪家寨、玉舍等卫星城的规划修编。进一步加快市中心城区建设步伐，完成凉都大道建设，完成人民路中段的改造。加快水城河亮丽工程建设，尽快完成水城河北岸排污管网建设，开工建设水城河南岸排污管网。完成市文化科技中心、沃尔玛大型超市主体工程建设。加快双水、凤凰、荷城、德坞新区的建设，启动黄土坡和老城的旧城改造。积极支持水城钢铁（集团）公司、水城矿业（集团）公司和各大电厂等企业建设新的行政区、生活区。进一步树立“大城管”理念，加强城市管理和市政基础设施建设，实施城市环境“六整治”，大力开展创建全国文明卫生城市活动。积极推进社区建设。加强城市绿化工作，搞好环城林带建设。在加快市中心城区建设的同时，坚持以经济流向为主导的城镇化发展战略，加快发展六枝平寨、盘县红果两个次中心城市，支持郎岱、岩脚、响水、发耳等重点小城镇建设。

　　促进区域经济协调发展，提高区域经济影响力。继续联合毕节、黔西南共同争取将南贵昆经济区贵州西部经济带（毕水兴经济带）纳入省“十一五”规划，争取国家和省在重大基础设施、生态环境治理等方面的支持，特别是在能源发展方面形成产业集群，逐步提高产业集中度和专业化协作水平，增强区域经济综合竞争力。进一步加大经济强县（区）、经济强乡（镇）建设力度，支持条件好的地区加快发展。加快小康乡镇和小康村建设。在有条件的部分乡镇适时启动行政区划调整和撤乡建镇工作。切实做好并村的后续管理和指导服务工作。

　　大力推进生态立市，积极发展循环经济。扎实抓好以林业为主的生态建设，继续实施好退耕还林工程，完成20万亩造林和3.5万亩封山育林。切实搞好水土保持工作，加快推进石漠化治理。认真落实国家森林生态效益补偿政策，加大对退耕还林、荒山造林、天然林资源等现有林的管护力度，严禁乱砍乱伐，巩固造林成果。加强环境保护，严格执行新上项目环保“三同时”制度。在控制新污染源的同时，突出重点区域、重点行业、重点污染物，加大环境污染的治理力度。积极争取国家和省的支持，启动三岔河和拖长江综合治理工程。抓好矿产资源勘查开发秩序专项整治，严厉打击非法勘查开采，依法查处非法转让矿权、圈而不采、以探代采和破坏性开采行为，特别要坚持“打击与取缔并举”的方针，严厉打击非法开采煤炭资源的违法犯罪行为，严防私挖滥采死灰复燃；力争解决好煤层气的勘探权和开采权问题。积极推行生态型开采和边采边恢复模式。全面推进探矿权、采矿权招标拍卖挂牌出让，培育和规范矿权市场。加强土地资源的保护和合理利用，做好市、县、乡土地利用总体规划修编，强化土地用途管制，严格执行耕地占补平衡制度。加强土地执法监察，严厉查处非法占用和破坏基本农田的行为。坚持开发和节约并举，把节约资源放在首位，在生产、流通、消费各个领域和各个环节，大力开展节能、节水、节材、节地活动，增强节约意识，提高资源综合利用效率。积极发展循环经济，开展工业热能利用、城市中水利用、工业固体废物利用的研究，提高资源综合利用水平。认真落实《中华人民共和国清洁生产促进法》，争取在部分重点城镇、重点工业区域、重点行业和重点企业开展清洁生产试点。抓好坡上牧场风力发电项目的前期工作。积极调整城乡能源结构，提高城市燃气和农村沼气普及率。优化市中心城区能源消费结构，加快取缔市中心城区燃煤锅炉，大力提倡使用燃气等清洁能源。

　　四、深化经济体制改革，进一步扩大对内对外开放

　　加快推进国有企业改革。认真落实省人民政府《关于加快我省国有中小企业改革的意见》，坚持“宜退则退，宜股则股，宜破则破”的原则，以产权制度改革为核心，以发展混合所有制经济为重点，以最大限度保护职工合法利益为底线，全面推进国有中小企业改革。今年困难企业国有资本退出要有大的突破，国有商贸企业改革要基本完成，国有建筑企业的改革要加快推进。切实抓好水城县烟叶复烤厂的关闭破产工作，尽快完成六盘水市三江商贸有限公司的划转。加快建立和完善国有资产管理体制和监管机制，着力理顺行业主管部门与国有资产管理部门的关系，切实推进“地企共建”。积极探索理顺市、县中小企业管理体制的有效途径。

　　坚持“两个毫不动摇”，鼓励、支持和引导非公有制经济加快发展。积极探索引导推动全市非公有制企业进行体制、机制、管理和技术创新的有效形式，促进非公有制企业经营规模不断壮大、经营管理水平和经营效益不断提高。积极构建非公有制经济服务体系，进一步发挥中小企业担保基金的作用，研究建立非公有制企业创业发展基金，不断完善和落实促进非公有制经济发展的政策措施，大力优化各种所有制经济共同发展的政策环境、政务环境、法制环境、市场环境、舆论环境。进一步放宽市场准入，支持和鼓励非公有资本进入基础设施、垄断行业、公用事业以及法律法规未禁止的其他行业。加大对损害非公有制经济发展事（案）件的查处力度。

　　进一步推进财政体制等改革。全面推行市级国库集中支付，进一步扩大县级国库集中支付实施面；全面实行政府采购，进一步完善和规范政府采购制度；全面实行乡镇财务“零户统管”，抓好“乡财县管乡用”和“村帐托管”试点。加强税收征管，加大对涉税犯罪的打击力度。加强煤炭企业煤炭产品税费征收管理，有效堵塞流通环节的跑冒滴漏。加强政府非税收入管理，继续深化“收支两条线”改革。在进一步做好农村税费改革的同时，配套推进乡镇机构、农村义务教育管理体制改革，组织开展乡村债务清理工作。加大国有粮食购销企业产权制度改革力度。加快城市信用社向商业银行发展的步伐，支持农村信用社加快改革步伐，力争引进交通银行入驻我市。继续推进投资体制改革。加快推进水利工程管理体制改革。

　　继续实施开放带动战略，不断扩大对外开放。积极开展与“泛珠三角”等区域的经济技术合作和交流，继续加强与湛江等8个友好城市的友好往来，大力推动对口帮扶城市大连市与我市广泛开展项目合作。积极组团到国外、东部发达地区和港澳台地区开展商贸洽谈、技术引进和招商引资等活动。发挥我市在川、滇、黔、桂结合区域和贵州西部经济带中的区位优势，进一步加强与周边地区的经济协作和工作协同。加快建立改善投资环境的长效机制，抓好以政府信用为核心的信用体系建设，积极建设“诚信六盘水”。严格执行损害投资软环境责任追究制度，抓住典型案例，加大查处力度。

　　五、统筹经济和社会协调发展，大力发展各项社会事业

　　坚持实施“科教兴市”战略和人才强市战略。一是全力抓好以“两基”为重点的教育事业，全面推进素质教育。进一步巩固提高盘县和钟山区的“两基”工作成果，切实抓好六枝特区、水城县的“两基”工作，确保通过省政府的评估验收。解决好“两基”攻坚后出现的寄宿制学校建设滞后、县乡教育投入不足、高中办学压力加大、民办教育规模小等问题。加快发展高中阶段教育，争办省级示范性高中，启动市一中双水校区、市三中凤凰校区建设。加快高等教育和职业技术教育发展步伐，完成六盘水师范高等专科学校教学主楼主体工程，建成六盘水职业技术学院综合楼主体工程。组织好贫困大学生助学贷款发放工作。搞好城乡学校布局规划，加快市中心城区教育资源整合步伐。切实管好用好教育费附加等教育资金，确保农村税费改革中央财政转移支付资金的50%用于教育基础设施建设，并不得用于发放教师工资。研究制定优惠政策，促进民办教育发展。大力实施农村中小学校现代远程教育工程。二是加快科学技术在生产领域的引进转化和推广应用。加大对农业科技成果转化的支持力度，着力扶持一批科技示范户、科技示范村。支持开展以企业为主体的技术创新体系建设，鼓励企业利用新技术、新工艺进行技术改造。积极组织开展适合我市的适用技术研究工作，进一步推进知识产权保护工作。三是加大人才工作力度。进一步落实好我市人才强市战略的有关政策，培养、引进、用好高层次和高技能人才，推进人才合理流动。注重乡土人才特别是农业科技人员的培养。

　　进一步抓好人口和计划生育工作，不断加强卫生工作。建立健全经常性工作机制，全面推进“少生快富”工程及“村为主”、“村（居）民自治”，进一步完善对农村计生家庭的奖励扶助制度，强化流动人口管理。坚持控制人口数量与提高人口质量并重，全面实施“三大工程”，综合治理出生人口性别比失衡问题。完善督查追究制，提高计划生育“两个及时率”。确保人口出生率控制在15‰以下，符合政策生育率保持在89%以上。加强公共卫生体系、疾病预防控制体系和医疗救助体系建设，积极推进医疗机构改革，强化卫生执法监督。积极开展城镇卫生体制改革，搞好新型农村合作医疗制度试点。坚持抓好地方病防治工作，落实重大传染病和地方病的防治措施。开展市中心城区医疗资源整合的有关前期工作。

　　加快繁荣文化体育事业，大力推进精神文明建设。认真做好历史文化遗产和地质遗迹的保护、开发和利用工作，加强对新民古生物化石、大洞古人类遗址及古建筑保护，开展毛口抢救性考古发掘、阿勒河大硝洞考古调查、古驿道考古调查等工作。加快县（特区、区）图书馆、文化馆建设，开工建设市文化科技中心二期工程。加大网吧治理整顿工作力度。继续举办“凉都?消夏文化节”和“金秋乌蒙”系列活动。加强民族民间文化的保护、抢救和发展，支持《夜郎王》电视剧的拍摄，加快布依族文化风情园建设，尽快完成苗族文化风情园的前期工作。加快发展体育事业，积极开展丰富多彩的体育活动，启动市中心城区新体育中心的规划编制工作。加强“盘县会议”会址等爱国主义教育基地的保护，大力弘扬以爱国主义为核心的民族精神、以艰苦创业为核心的“三线建设”精神、以改革创新为核心的时代精神。开展抗日战争胜利60周年纪念活动。积极推进群众性精神文明创建活动，大力倡导科学健康文明的生活方式。加强对青少年的思想道德教育。深入开展以“情系农家、富裕农民、美化农村”为主题的农村精神文明创建活动。积极引导先富起来的群体增强社会责任感。

　　六、加强社会建设和管理，积极构建社会主义和谐社会

　　继续做好就业再就业和社会保障工作。大力实施积极的就业政策，通过发展多种所有制经济和劳动密集型产业，拓宽就业渠道，增加就业岗位。进一步落实优惠政策，加强就业培训，搞好就业指导和服务。统筹做好高校毕业生、复员转业军人、城镇新增劳动力和农村剩余劳动力就业工作，促进就业1.8万人，其中促进下岗失业人员再就业4800人、安置“4050”人员600人。进一步加快建立和完善社会保障体系，继续巩固“两个确保”，做好“三条保障线”的衔接，进一步加强城乡社会救助体系建设。规范和完善城市低保工作，认真落实城市低保对象享受的有关优惠政策和待遇。加快推进工伤保险，争取完成10万人的参保任务。

　　认真解决事关群众切身利益的突出问题，妥善安排好困难群众的生产生活。认真落实国务院《关于深化改革严格土地管理的决定》，依法完善征地补偿办法，防止出现“程序不规范，补偿不到位，安置不落实，方法不得当”等问题，坚决防止和纠正土地征用和房屋拆迁中侵害城乡居民利益的现象。高度重视失地农民的长远生计问题，积极探索失地农民参加养老、医疗等社会保险的思路，切实抓好盘县响水“一厂一矿”征地拆迁失地农民长远生计的试点工作，在省有关部门的支持下启动钟山区荷城办事处大海坝市场安置试点工作。认真落实有关政策规定，坚决纠正企业重组改制和破产中侵害职工合法权益的问题。强化对企业工资特别是企业管理层工资的监控。进一步加大劳动保障执法监察力度，坚决制止拖欠和克扣农民工工资，坚决打击恶意克扣农民工工资行为。探索建立解决拖欠民工工资问题的长效机制，确保在今年年底前完成政府投资工程拖欠工程款的偿还、2006年年底前完成其他社会工程拖欠工程款的偿还并防止发生新的拖欠。加大治理教育乱收费力度，全面推行义务教育阶段“一费制”的收费办法。加强对药品集中招标采购的管理和监督，切实纠正医药购销和医疗服务中的不正之风。积极发展社会福利事业，加快完善农村五保户供养、特困户救助和医疗救助制度，开展钟山区城市特困救助体系建设试点。进一步完善措施，切实解决好农村生产生活用煤问题。积极理顺收入分配关系，在省启动的基础上适时启动市级机关“阳光补贴”工作。

　　高度重视安全生产和灾害防治工作，保障国家和人民生命财产安全。认真落实2005年市委“一号文件”，进一步做好基层和基础工作，落实生产经营单位安全生产责任主体和投入主体地位，采取政策、法律、经济和行政的综合治理措施，切实解决安全生产工作中“严不起来，落实不下去”的问题，真正“严起来、硬起来、狠起来”。认真落实救灾工作体制，增强自然灾害紧急救援能力。切实把地质灾害防治作为一项长期而紧迫的任务来抓，加快完成《六盘水市地质灾害防治规划》的编制，建立地质灾害预警预报系统，认真落实地质灾害防治预案。继续搞好地质灾害特别危险点的搬迁工作，完成500户地质灾害点移民搬迁任务。争取在国家的支持下启动采煤沉陷区地质灾害治理工程。

　　切实维护社会稳定。进一步建立健全人民调解工作新机制，积极化解民间纠纷，把不稳定因素消除在基层和萌芽状态。健全正确处理人民内部矛盾的工作机制，认真做好人民群众来信来访工作，依法及时合理地处理群众反映的问题。高度重视对群体性事件的预防，妥善处置群体性事件。继续整顿和规范市场经济秩序。进一步深化警务体制改革，扎实抓好创建“平安六盘水”工程，不断加强社会治安综合治理；依法严厉打击各种刑事犯罪特别是黑恶势力犯罪、严重暴力犯罪；加大禁毒工作力度，严厉打击赌博违法犯罪活动，维护正常的社会秩序。

　　在重点抓好以上工作的同时，要统筹兼顾地做好审计、统计、物价、档案、外事、侨务、对台等工作；全面贯彻党的民族宗教政策；加强国防教育，抓好民兵预备役队伍建设，广泛深入开展“双拥”共建活动；支持工会、共青团、妇联等人民团体依照章程开展工作。按照“积极、科学、加快、向上”的原则，认真制定好全市“十一五”发展规划，强化规划引导发展的意识，严防建设不符合规划要求的项目，确保规划的严肃性。

加强政府行政能力建设

　　加快转变政府职能，按照科学执政、民主执政、依法执政的要求，突出制度建设，进一步完善政府工作制度，切实做到有章可循、按章办事、依法行政，不断规范从政行为和提高行政效能，不断提高领导发展的能力和水平，不断适应新形势下加快发展的需要。

　　一是要切实转变政府职能。进一步深化行政管理体制改革，全面履行经济调节、市场监管、社会管理、公共服务四大职能，更加重视政府的社会管理和公共服务职能，集中精力解决好社会进步和国计民生问题，做到不缺位、不越位、不错位。切实增强全面落实科学发展观、按市场经济规律管理经济、运用经济和法律手段调节经济运行的意识和能力。加快建立健全各种突发事件应急机制，提高保障公共安全和处置突发事件的能力。今年，各级政府要围绕自然灾害、事故灾难、公共卫生和社会安全等方面，尽快制定《突发公共事件总体应急预案》和专项应急预案并认真组织演习，切实贯彻执行。加快电子政务二期工程建设，创新政府管理方式。

　　二是要提高依法执政水平。围绕建设法治政府的目标，切实贯彻落实国务院《全面推进依法行政实施纲要》，全面推进依法行政。认真贯彻执行《中华人民共和国行政许可法》，减少和限制不必要的行政审批事项和于法无据的事项。加强配套制度建设，落实行政审批制度改革后续监管措施。加大对行政审批中违纪违规行为的责任追究力度。完善行政执法机关的内部监督制约机制。建立公开、公平、公正的评议考核制和执法过错或错案责任追究制。建立健全规范性文件修改和废止的工作制度以及规范性文件的定期清理和定期评价制度。推进“四五”普法进程，搞好“五五”普法规划。

　　三是要大力推进决策的科学化和民主化。认真研究界定各级政府、政府各部门的行政决策权，完善政府内部决策规则、决策程序以及行政决策的监督制度和机制。按照“谁决策、谁负责”的原则，建立健全决策责任追究制度。建立健全公众参与、专家论证和政府决定相结合的行政决策机制。建立和完善重大问题集体决策制度、专家咨询制度、社会听证制度和决策责任制度。自觉接受同级人大及其常委会的法律监督和工作监督，自觉接受同级政协的民主监督，认真听取各民主党派、工商联、无党派人士和人民团体的意见，自觉接受新闻舆论和社会公众监督。

　　四是要大力加强政风建设。严格按照中央确定的指导思想、目标要求、基本原则和省委、市委的统一部署，认真开展保持共产党员先进性教育活动。加强行政效能建设，大力推行首问负责制。按照“有权必有责、用权受监督、侵权要赔偿”的要求，加快建立问责制度。坚持“便捷、高效、透明、为民”的原则，进一步加强政务公开的监督、监管工作，扩大群众的知情权、参与权、监督权。建立健全政府新闻发言人制度，实现政府与媒体、公众的及时沟通。建立政府信息公开制度，方便公众对政府信息的获取和查阅。按照树立正确政绩观的要求，加快建立和完善政绩考核和评价体系。不断强化宗旨观念，继续开展“三满意”创建活动，加快公共服务中心（行政服务中心）建设。改进会风和文风，大力精简会议和文件，大兴调查研究之风。切实为群众办实事、办好事。认真办好2005年“十件实事”，真正取信于民。全面贯彻标本兼治、综合治理、惩防并举、注重预防的方针，按照中央颁布的《建立健全教育、制度、监督并重的惩治和预防腐败体系实施纲要》的要求，教育和督促政府系统的各级领导干部加强党性修养，牢记“两个务必”，自觉做到“八个坚持、八个反对”。认真贯彻落实中纪委、省纪委五次全会和国务院、省政府廉政工作会议精神，全面推进政府廉政建设和反腐败工作。切实树立“细节决定成败”的意识，加强对各项工作的全过程跟踪，对每个环节和细节进行适时监督，保证工作落到实处。

　　各位代表：做好今年的各项工作，任务艰巨，责任重大，前景美好，使命光荣。让我们紧密团结在以胡锦涛同志为总书记的党中央周围，高举邓小平理论和“三个代表”重要思想伟大旗帜，用胡锦涛同志在贵州视察工作时的重要讲话精神统领各项工作，抢抓机遇，用好机遇，为全面完成和超额完成今年经济社会发展的各项任务和“十五”计划目标，为尽快实现我市经济社会发展的历史性跨越而努力奋斗！
